# Miscellaneous Notices

**Published:** 1841

**Authors:** 


### MISCELLANEOUS NOTICES.

The following notices were intended for the August and September Nos. of the Journal, but were unavoidably excluded for the want of room.

*Prospectus of the Facultyof Physic in the University of Maryland.*

We find that the vacancy in the Surgical Chair of this old and highly respectable School, is filled by the appointment of Dr. N. R. Smith, formerly professor in the institution. Dr. Smith needs no commendation from us. His value is too well known to the public to be enhanced by our attestation. As a Surgeon, and as a teacher of Surgery, Professor Smith has few equals. The other Chairs are filled as they were last year.

The Professors are about to undertake great additional labour by lecturing during a term of six, instead of four months. We hope that this innovation may turn out to be reform. The Lectures commenced on the first of September.

—

*Annual Circular of the Washington University, of Baltimore—Medical Department.*

This institution has been quietly growing in importance. The Professors have perseveringly laboured to deserve success rather than to secure it. They have spent more time in instructing their pupils than in puffing themselves, and consequently have advanced the interests of their School steadily though slowly.

The buildings of the Washington University, are, in our opinion, better adapted to their purposes than any similar buildings in the United States. Connected with commodious lecture rooms, &c., are a most excellent hospital and rooms for the accommodation of a number of resident students. These board in the house, and have great advantages.—Were we a student again, we should board in the Washington College, and learn practical knowledge at the bed-side of a hundred patients, rather than lodge in a crowded boarding house in a city, where seclusion for study is impossible, and learn practical knowledge no where. We like this plan of teaching students their business. We are convinced that many a graduate in medicine, who has never seen a sick person, goes to the practice of his profession with a sickening sense that he is utterly unqualified to distinguish and prescribe for disease. A man can as easily learn seamanship from books as he can the practice of physic.

—

*Hamden Sidney College—Richmond, Va.*

From our Knowledge of the Professors in this School, students may expect courses of Lectures equal to any that will be delivered elsewhere.

The Session will commence on Monday, 2d November. We are well satisfied to see that Virginia is now awake to the importance of educating her own physicians. We care not how many Medical Schools are established, provided they limit their competition to a struggle for excellence, and not for popularity.

---

*Advancement of Medical Science—Warsaw University of Illinois.*

We learn with pleasure that this important institution is, by its Charter, authorized to, and has established a Medical Department. From our knowledge of the President, Doct. J. B. Perry, and the gentlemen associated with him, we have no doubt that this infant institution will flourish and do good. Its establishment reflects the highest credit upon the State, and shows that she is alive to the all-important subject of education; and that in the cultivation of Literature and the Sciences, she is determined not to be behind other sections of the country.

We have read with much pleasure, the inaugural address of the President. It is an able production. The importance of education is set forth in it, in a clear and masterly manner.

